# Radioiodine treatment outcome by dosimetric parameters and renal function in hyperthyroidism

**DOI:** 10.1186/s13044-022-00126-4

**Published:** 2022-04-25

**Authors:** Joachim N. Nilsson, Rebecca Elovsson, Daniel Thor, Jan Calissendorff, Oscar Ardenfors

**Affiliations:** 1 Department of Molecular Medicine and Surgery, Karolinska Institutet, 17176 Stockholm, Sweden; 2 Department of Medical Radiation Physics and Nuclear Medicine, Karolinska University Hospital, Stockholm, Sweden; 3 Department of Oncology-Pathology, Karolinska Institutet, Stockholm, Sweden; 4 Department of Endocrinology, Metabolism and Diabetes, Karolinska University Hospital, Stockholm, Sweden

**Keywords:** Hyperthyroidism, Radioiodine therapy, I-131, Dosimetry, Renal function

## Abstract

**Background:**

Hyperthyroidism has been treated with radioiodine therapy for eight decades, with known benefits and side-effects. No consensus exists on which activity dosage and pre-therapeutic measurements are required for optimal treatment, balancing risk of incomplete response, therapy-induced hypothyroidism and radiation exposure. A retrospective analysis was performed to assess these questions.

**Methods:**

Data was collected on radioiodine treatment outcomes for 904 patients treated for Graves' disease or toxic nodular goitres at our institution during 2016–2020. The prescribed absorbed doses were 120 Gy (Graves’ disease), 200 Gy (toxic multinodular goitre) and 300 Gy (solitary toxic adenoma). Univariate analysis and multivariate regression modelling were used to find factors linked to treatment outcome.

**Results:**

The cure rate of hyperthyroidism after one administration of radioiodine was 79% for Graves' disease, 94% for toxic multinodular goitre and 98% for solitary toxic adenoma. Thyroid mass, uptake and effective half-life were all significantly associated with cure in Graves’ disease, but not in toxic multinodular goitre. The rates of therapy-induced hypothyroidism were 20% and 29% for toxic multinodular goitre and solitary toxic adenoma. Neither the cure rate nor the hypothyroidism rate was found to be superior among patients with individualised effective half-life measurements in toxic nodular goitres. Poor renal function was associated with dubious iodine uptake measurements but was not found to correlate with worse outcome.

**Conclusions:**

Multiple measurements of individual iodine uptake for kinetics estimation may be unnecessary, and a population-based value can be used instead. Patients with renal impairment had similar outcome as other patients, but with a higher risk of dubious uptake measurements.

**Supplementary Information:**

The online version contains supplementary material available at 10.1186/s13044-022-00126-4.

## Introduction

Hyperthyroidism is a condition in which the thyroid gland produces excessive amounts of thyroid hormones disturbing the body metabolism, potentially leading to a substantial risk increase of cardiovascular disease and a decrease in quality of life if it remains untreated [1, 2]. The most common types of hyperthyroidism are Graves’ disease (GD) and toxic nodular goitre (TNG) (comprising toxic multinodular goitre (TMNG) and solitary toxic adenoma (STA)). The annual incidence rate for those conditions combined is roughly 50 per 100,000 in Europe [3, 4]. Hyperthyroidism is typically treated with either medication using antithyroid drugs, radioactive iodine (iodine-131), or surgery. Radioiodine has been used for eighty years to treat hyperthyroidism and is considered a safe, easily performed and cost-efficient treatment.

When treating GD with radioiodine, the aim is to completely irradiate the thyroid gland, resulting in a hypothyroid state in the patient [5]. For TNG, the aim is to quench the hyperactive nodules by localised irradiation, thereby making the patient euthyroid [6]. As therapy-induced hypothyroidism requires patients to be prescribed hormone substitution, the number of treated patients suffering from hypothyroidism in the TNG group should be kept as low as possible without severely impairing the cure rates of hyperthyroidism. Hypothyroidism can occur several years post treatment and patients treated with radioiodine should consequently be monitored for a long follow-up period [7, 8].

The prescribed absorbed dose (in Gy) in radioiodine treatments of hyperthyroidism is usually based on the disease subtype and determines the amount of activity (in MBq or mCi) to be administered to the patient. The relation between activity and absorbed dose is patient-specific and parameters such as thyroid iodine uptake, effective half-life and mass are sometimes used to individually calculate the amount of administered activity [9]. Due to practical and economic reasons, it is also common to use fixed activities for specific prescribed doses, not taking patient kinetics into account [10]. If the relation between activity and dose is inaccurate, e.g. due to reduced kidney function, the patient may be over- or undertreated [11, 12]. Overtreatment can manifest as initial hypothyroidism which has reported incidence rates of 10–20% in patients treated for STA [13]. On the other hand, recent data showed that 18% of patients with GD were not cured after their first radioiodine treatment, indicating a degree of under-treatment [5]. The number of over- and undertreated patients could potentially be reduced by an increased accuracy in administered activity calculations. This is further motivated by the fact that all unnecessary dose contributions should be reduced as they may incur an increased risk of radiation-induced cancer, although this is debated [14, 15].

The aim of this work was to evaluate, in a large cohort, the rate of cure and therapy-induced hypothyroidism. We analysed the necessity of measurements of thyroid iodine uptake and effective half-life in the calculation of administered activity. We also wanted to investigate the efficacy of treatment in the patients with impaired renal function.

## Methods

A total of 904 patients diagnosed with hyperthyroidism and treated with their first radioiodine treatment during the years 2016–2020 at Karolinska University Hospital were included in this study. The patients were diagnosed with either GD, TMNG or STA. Patient data was obtained from medical records and the treatment outcome was acquired and evaluated. Treatment outcomes were related to patient-specific parameters such as administered activity, patient age, thyroid mass, effective half-life, renal function (as estimated glomerular filtration rate, eGFR), serum thyroid stimulating hormone (TSH) prior to treatment and absorbed dose. The patient parameters are summarised in Table 1.

**Table 1:** Summary of collected patient data presented as median values and (interquartile ranges)

Parameter	All diagnoses	Graves’ disease	Toxic multinodular goitre	Solitary toxic adenoma
No. of patients	904	335	394	175
Age [years]	66 (52 75)	53 (44 67)	72 (64 78)	66 (55 75)
Females [%]	84	82	85	84
Uptake at 24 h [%]	37 (28 53)	57 (45 65)	31 (25 38)	31 (25 38)
Target mass^a^ [g]	45 (30 55)	50 (45 55)	45 (35 55)	25 (20 30)
Absorbed dose [Gy]	-	123 (120 125)	176 (130 202)	293 (237 305)
Activity [MBq]	-	447 (370 756)	805 (709 825)	805 (712 828)
Effective half-life^b^ [d]	6.4 (5.8 7.2)	6.2 (5.4 6.9)	6.9 (6.3 7.4)	6.3 (5.7 6.8)
eGFR [ml/min/1.7 m^2^]	75 (63 89)	83 (70 90)	71 (58 81)	76 (66 87)

^a^Mass of hyperactive thyroid tissue

^b^Determined for 392 patients who underwent uptake measurements at 24 h and 7 d post administration of tracer amounts of iodine-131

### Radioiodine therapy

**Fig. 1:**
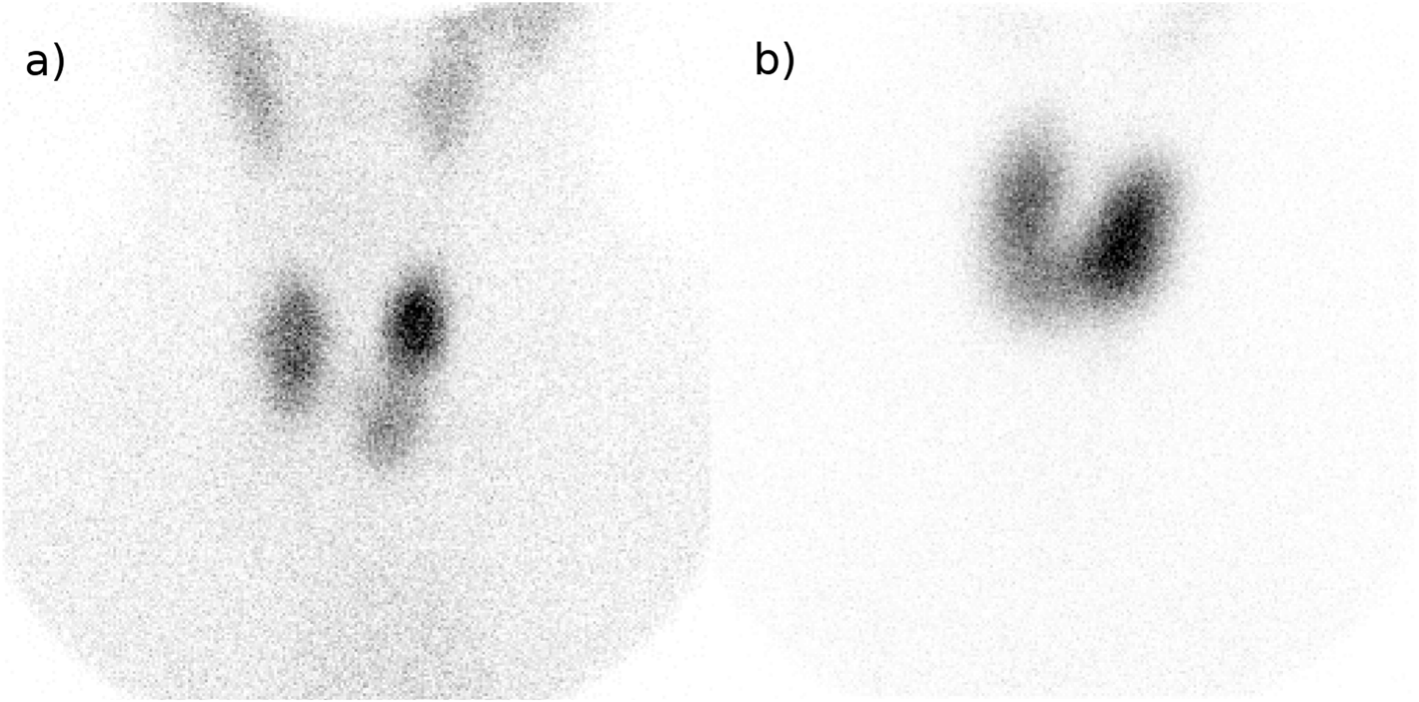
Planar scintigraphy of **a**) solitary toxic adenoma and **b**) Graves' disease

All patients received radioiodine therapy with an amount of activity calculated according to Eq. 1, which was derived from [16]:

1 $$A=\frac{D\times m\times 23.3}{{U}_{24}\times {T}_{1/2}}$$

where *A* denotes administered activity of iodine-131 [MBq], *D* denotes prescribed absorbed dose [Gy], *m* denotes hyperactive thyroid mass [g], *U*_*24*_ denotes the thyroid iodine uptake at 24 h [%], and *T*_*1/2*_ denotes the effective half-life of iodine in the thyroid [d]. The mass of the hyperactive thyroid tissue was determined from ellipsoid volume calculations based on hyperactive nodules (TNG) or full thyroid volume (GD) on technetium-99 m planar scintigraphy. Typical unfiltered scintigraphies used as clinical routine in this cohort are shown in Fig. 1.

Ultrasound is not routinely used at our institution due to difficulties in estimating hyperfunctional volume in patients with TNG and ultrasound images were therefore not included in the analysis [17]. The effective half-life of the thyroid was calculated from NaI-scintillator uptake measurements at 24 h and 7 d post ingestion, assuming a mono-exponential uptake curve. While calculation of the effective half-life has been the standard procedure at our institution, if logistical or medical reasons prohibited a measurement of iodine uptake at 7 days, the administered activity was calculated using a pre-determined fixed effective half-life of 5.3 days for GD or 6.5 days for TNG. The prescribed absorbed doses were 120 Gy (GD), 200 Gy (TMNG) and 300 Gy (STA). These dose levels have evolved at our institution over time, but they are somewhat in line with published procedure guidelines, for example from the European Association of Nuclear Medicine [7]. If the calculated activity exceeded 800 MBq, the patient was administered only 800 MBq. This caused some individually delivered absorbed doses to be lower than the prescribed absorbed dose for TMNG and STA. Levothyroxine and antithyroid drugs were discontinued 7 days before radioiodine treatment. If patients had severe renal impairment (eGFR < 30 ml/min/1.7m^2^), the prescribed absorbed dose was reduced by up to 80%.

### Treatment outcome

**Fig. 2:**
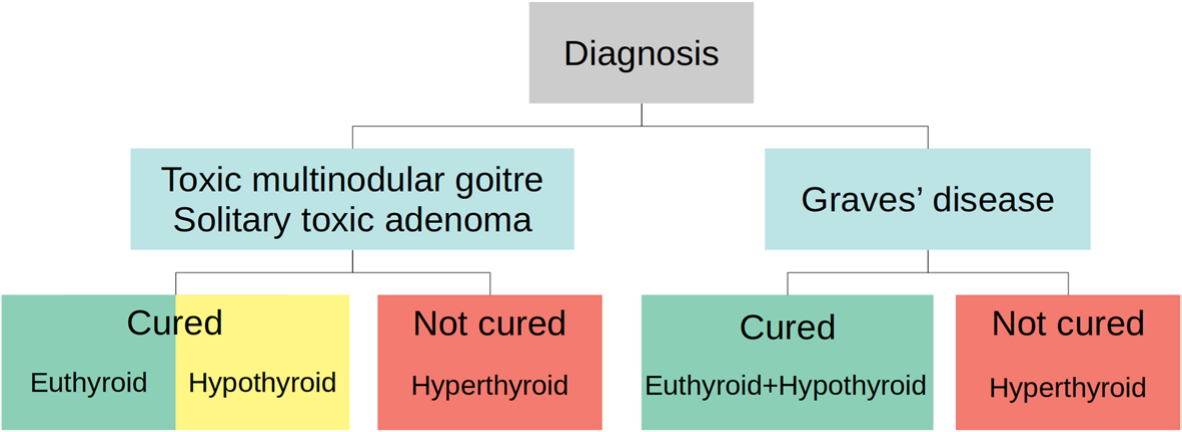
Illustration of how treatment outcomes were categorised. The number of patients for each diagnosis and outcome rates are reported in Table 2

The treatment outcomes were divided into different categories depending on diagnosis according to Fig. 2. Follow-up was at least three months, after which patients cured from hyperthyroidism were referred to their primary care provider. If patients had persistent hyperthyroidism, follow-up was continued by the Department of Endocrinology, Metabolism and Diabetes at Karolinska University Hospital. Euthyroid (no levothyroxine prescription at follow-up) and hypothyroid post-treatment states were analysed separately for TNG patients. For patients with GD, both euthyroid and hypothyroid states were considered cured, regardless of prescription status of levothyroxine. At our institution, gradual levothyroxine substitution shortly after radioiodine treatment is used for most patients with GD. We were therefore unable to distinguish rates of hypothyroidism from euthyroidism at three months follow-up for GD in our cohort.

### Effective half-life estimation

Analyses were carried out to investigate the influence on treatment outcome by effective half-life estimation method. Treatment outcomes were compared between patients who were either treated with a prescribed activity determined from patient-specific uptake measurements at 24 h and 7 d, or by using fixed effective half-life values (394 patients). The treatment outcome of all patients was analysed separately for each diagnosis.

### Renal Function

Impaired clearance of iodine caused by poor renal function may delay the uptake phase of iodine into the thyroid and thereby shift the thyroid uptake curve [12]. Renal function was included in the analysis to analyse the implications of this effect. A delayed thyroid uptake with maximum uptake occurring later than 24 h post ingestion will result in erroneous effective half-life calculations when assuming a mono-exponential uptake curve. This can manifest as effective half-lives estimated to more than the physical half-life of iodine-131 (8 days). The eGFR was analysed with respect to treatment outcome, and to the effective half-life, to investigate if a delayed iodine uptake in the thyroid could be linked to reduced renal function. Thus, the eGFR values in patients with calculated effective half-lives > 8 d (118 patients) was compared with the eGFR values of all other patients.

### Statistical analysis

The statistical analysis was carried out using R version 3.6.3 (R-project.org). Differences in continuous variables between dichotomous groups (i.e. cured/not cured, euthyroid/hypothyroid outcome) were analysed using Welch’s t-test. Differences in cure rate between dichotomous groups were analysed using Pearson's chi-squared test. Multivariate logistic regression models were created to investigate potential predictor variables and confounding variables with respect to treatment outcome. The logistic regressions were performed for each diagnosis and used the input parameters listed in Table 1. The model was tested for multicollinearity using variance inflation factors. Heteroscedasticity was assessed using Goldfeld-Quandt tests, and the distribution of residuals was studied by inspection of histograms. The models were optimised using a backwards directed Akaike Information Criterion (AIC) stepwise algorithm to reduce model complexity. Due to the exploratory nature in finding predictor variables in this work, confidence levels were only considered statistically significant for *p* < 0.01.

## Results

### Treatment outcome

The overall treatment outcomes for all studied diagnoses are presented in Table 2. The results show a lower cure rate for patients with GD compared to patients with TNG. The cure rate of initial radioiodine treatment of GD was 79%. However, most of the GD patients with unsuccessful treatment in this group (50 of 73) received a second radioiodine treatment, which cured 49 out of 50. The other patients (23 of 73) were treated with surgery, antithyroid drugs or were still in follow-up during study data collection. For TMNG and STA, 94% and 98% of patients were cured of hyperthyroidism after one initial radioiodine therapy, and 20% and 29% of these patients developed therapy-induced hypothyroidism within three months. Mean values of the collected parameters, with their associated statistical significance, for cured and not cured patients are presented in Table 3. The variables with statistical significance for predicting cure in GD were thyroid uptake of iodine at 24 h, thyroid mass and effective half-life. In the multivariate model for cure of GD, thyroid uptake of iodine at 24 h and effective half-life were found to be statistically significant. The optimised logistic models all had low variance inflation factors. However, the model for TMNG had problems with heteroscedasticity, and was therefore not considered reliable and not used in the final analysis. All models had some degree of non-normally distributed residuals, but less so in the case of GD.

**Table 2:** Treatment outcome for patients treated with one administration of radioiodine for Graves’ disease, toxic multinodular goitre and solitary toxic adenoma

	All diagnoses	Graves’ disease^a^	Toxic multinodular goitre	Solitary toxic adenoma
No. of patients	904	335	394	175
Cured [%]	89.3	78.5	94.4	98.3
Euthyroid			74.6	69.1
Hypothyroid			19.8	29.1
Not cured [%]	10.7	21.5	5.6	1.7

^a^No euthyroid/hypothyroid discrimination due to aim of treatment stated inMethods

**Table 3:** Patient parameters of cured (euthyroid and hypothyroid) and not cured patients for Graves’ disease, toxic multinodular goitre and solitary toxic adenoma. *p*-values of t-test is displayed

		Graves’ disease		Toxic multinodular goitre		Solitary toxic adenoma	
Parameter	Outcome	Mean	p	Mean	p	Mean	p
Age [years]	Cured	55	0.28	70	0.04	63	0.68
	Not cured	53		72		66	
24 h uptake [%]	Cured	53	< 0.01*	32	0.14	32	0.70
	Not cured	60		37		29	
Mass [g]	Cured	49	< 0.01	46	0.64	26	0.48
	Not cured	54		50		45	
Abs. dose [Gy]	Cured	122	0.38	162	0.45	266	0.32
	Not cured	123		152		192	
Activity [MBq]	Cured	477	0.01	764	0.01	757	0.80
	Not cured	434		630		737	
Eff. half-life [d]	Cured	6.0	< 0.01*	6.8	0.81	6.2	-
	Not cured	6.5		6.5		§	
eGFR [ml/min/1.7m^2^]	Cured	78	0.34	70	0.02	74	-
	Not cured	80		57		§	
Pre-treatment TSH (mU/l)	Cured	0.89	0.06	0.16	0.24	0.07	0.39
	Not cured	0.49		0.10		0.12	

^§^No eGFR-data was available due to small number of patients

^*^Significant (*p* < 0.01) in multivariate logistic regression model

### Effective half-life estimation

Cure rates for patients who were treated with activities calculated using patient-specific effective half-lives are presented in Table 4 together with cure rates for patients who were treated with activities based on fixed effective half-lives. The measured effective half-lives were 6.1 d, 6.8 d and 6.2 d for GD, MTG and STA respectively, compared to the fixed half-lives of 5.3 d, 6.5 d and 6.5 d in use at our institution. The results show that the probability of being cured was significantly higher when a fixed effective half-life was used for patients with GD while no difference was found for TNG.

**Table 4:** Cure rate for patients treated with activities based on patient-specific effective half-lives or with activities based on fixed effective half-lives

Diagnosis	Patient-specific half-life	Fixed effective half-life	*p*-value
Graves’ disease	71%	85%	< 0.01
Toxic multinodular goitre	96%	94%	0.99
Solitary toxic adenoma	100%	97%	0.95

### Renal function

**Fig. 3:**
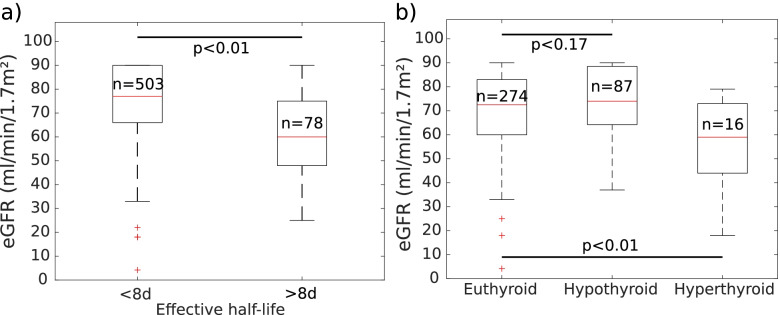
**a**) Estimated effective half-lives of less than 8 days is plotted against patients with suspected erroneous half-life estimations (> 8 days). **b**) eGFR for patients with TNG, who became euthyroid, had persistent hyperthyroidism or developed therapy-induced hypothyroidism

The hypothesis that low eGFR causes erroneous uptake measurements was tested by comparing eGFR among patients that had obviously incorrectly estimated effective half-lives of > 8 days. The cohort included patients with eGFR in intervals > 90 ml/min/1.7m^2^ (143 patients, 25%), 60–90 ml/min/1.7m^2^ (321 patients, 55%), 30–60 ml/min/1.7m^2^ (110 patients, 19%) and < 30 ml/min/1.7m^2^ (6 patients, 1%). Patients with long effective half-lives (> 8 days) had lower eGFR than patients with shorter half-lives (*p* < 0.01), as displayed in Fig. 3a. This indicates that reduced renal function increases the risk of inaccurate uptake measurements—and subsequently inaccurate absorbed dose calculations based on such data. The renal function in patients with TNG was lower in those with persistent hyperthyroidism than in those who became euthyroid (*p* = 0.01). Patients that had persistent hyperthyroidism and renal impairment (< 30 ml/min/1.7m^2^) were treated with an 80% reduction of prescribed dose, which may in part explain this difference. Renal function of patients with TNG who developed therapy-induced hypothyroidism was not significantly different to that of patients who achieved a euthyroid post-therapy state (*p* = 0.17), as can be seen in Fig. 3b.

## Discussion

The treatment cure rate was 94% and 98% for TMNG and STA, respectively. It was found that 20% and 29% of patients with TMNG and STA developed hypothyroidism within a follow-up period of three months. These rates of TNG cure and therapy-induced hypothyroidism are in the higher range of previously published data [18–22]. Comparing administered activity in this investigation with other publications shows that our high cure rates may be due to an overall higher administered activity to patients with TNG (see supplementary material S1). It should be noted that the exact values of absorbed doses derived for the patient cohort in this study may not be applicable in other clinics due to variations in how parameters such as thyroid uptake and mass are determined and used in dosimetric calculations [23]. The impact on cure rate when using patient-specific or fixed effective half-lives was negligible in TNG, as seen in Table 4. The pre-determined fixed effective half-life of 6.5 d was in good agreement with the mean values of the measured effective half-lives of 6.8 d and 6.2 d for TMNG and STA.

Considering the very high cure rate of 99% for STA, the rate of therapy-induced hypothyroidism may be decreased, or at least delayed in its onset, by prescribing a lower absorbed dose to these patients. However, the rate of therapy-induced hypothyroidism seems less influenced by reductions in administered activity (see supplementary material S1). A limitation of the current work is the short follow-up time, as the occurrence of hypothyroidism in patients referred to their primary-care provider after cure of hyperthyroidism is expected to increase with time, as reported by others [5, 7, 8]. Another limitation in this work that should be considered when interpreting the results is the use of planar scintigraphy for target tissue delineation. Mass estimation from planar images and ellipsoid approximations are known to have a high variance depending both on the individual thyroid shape and the interpersonal differences in size estimation [24]. This may increase the variance in absorbed doses delivered and thereby obfuscate underlying dose–response relations.

The results for patients with GD showed that using a prescribed dose of 120 Gy resulted in a cure rate of 79%. These values are in line with previously published data on GD [5, 25]. Previously, dose–response correlations have been established for GD, and shown that an absorbed dose of 200 Gy would cure 80% of patients [26]. The results from the current work shows similar outcomes at a lower absorbed dose. A recent meta-analysis showed that absorbed doses of 120–180 Gy were most likely to cure patients and produce a euthyroid state in patients with Graves' disease [27]. However, dosimetric methodology can differ and may explain the discrepancy between studies and the multivariate logistic regression model in this work demonstrated that only thyroid uptake at 24 h was a predictor for cure in GD. When including patients with GD who were given a second administration of radioiodine, the cure rate was 99%, indicating that these patients were undertreated by the initial administration. It was demonstrated that the cure rate for GD was lower when a patient-specific value of the effective half-life was used (mean 6.1 d), compared to using a fixed half-life of 5.3 d. As follows from Eq. 1, patients with fixed effective half-lives therefore received higher absorbed doses than those with measured effective half-lives. Consequently, the value for fixed effective half-life used at our institution (5.3 d) was too low for the GD cohort; it was adjusted accordingly as a consequence of this study. This was likely a strong contributor to effective half-life being a statistically significant predictor of cure in GD. The results in this work confirms the influence of thyroid iodine uptake and thyroid mass as a strong predictor of cure [27].

Our results suggest that the use of fixed effective half-lives based on population averages can be cost-effective and produce successful treatment outcomes. While such practice appears to be fully adequate for most patients, it may be precarious for patients with uncommon thyroid iodine kinetics, which cannot be accounted for in a population-based value of effective half-life. It was demonstrated that uptake measurements and consequently, patient-specific half-lives were more likely to be incorrect for patients with reduced kidney function (Fig. 2a). This indicates that additional uptake measurements might be needed to achieve reliable measures of effective half-life in patients with low eGFR. However, the regression analysis in this work showed that patients with severe renal impairment did not have worse treatment outcome in general. The reduction of prescribed absorbed dose by up to 80% for patients with eGFR < 30 ml/min/1.7m^2^ employed at our institution was significantly associated with lower cure rate, albeit in a small group of six patients out of 904.

## Conclusions

The cure rates in this work were in line with reported values for GD from previous studies. The cure rate was substantially higher in patients with TNG compared to previously published data, possibly explained by higher prescribed absorbed doses at our institution. The treatment outcome for GD was better in patients who were treated with activities based on fixed effective half-lives instead of patient-specific measured values. Dubious uptake measurements were more common in patients with reduced kidney function. The findings of this study could help in finding the optimal balance between cure rate, therapy-induced hypothyroidism and radiation exposure.

## Supplementary Information

**Additional file 1: Table S1.** Comparison of outcome in the present study to articles in the literature that specified frequencies of eu-, hypo- and hyperthyroidism for both Nodular and Graves (nonsystematic review). The table is sorted by amount of activity. **Figure S2.** Graph of the data from Table 1 for the Graves cases.

## Data Availability

The study data files in RData and xlsx format are available upon reasonable personal request.

## Abbreviations

GD: Graves' disease
TNG: Toxic nodular goitre
TMNG: Toxic multinodular goitre
STA: Solitary thyroid adenoma
eGFR: Estimated glomerular filtration rate
TSH: Thyroid stimulating hormone

## References

[1] Taylor PN, Albrecht D, Scholz A, Gutierrez-Buey G, Lazarus JH, Dayan CM (2018). Global epidemiology of hyperthyroidism and hypothyroidism. Nat Rev Endocrinol.

[2] Jabbar A, Pingitore A, Pearce SHS, Zaman A, Iervasi G, Razvi S (2017). Thyroid hormones and cardiovascular disease. Nat Rev Cardiol.

[3] Garmendia Madariaga A, Santos Palacios S, Guillén-Grima F, Galofré JC (2014). The incidence and prevalence of thyroid dysfunction in Europe: a meta-analysis. J Clin Endocrinol Metab.

[4] Abraham-Nordling M, Byström K, Törring O, Lantz M, Berg G, Calissendorff J (2011). Incidence of hyperthyroidism in Sweden. Eur J Endocrinol.

[5] Sjölin G, Holmberg M, Törring O, Byström K, Khamisi S, de Laval D (2019). The Long-Term Outcome of Treatment for Graves’ Hyperthyroidism. Thyroid.

[6] Ross DS, Burch HB, Cooper DS, Greenlee MC, Laurberg P, Maia AL (2016). American Thyroid Association Guidelines for Diagnosis and Management of Hyperthyroidism and Other Causes of Thyrotoxicosis. Thyroid.

[7] Stokkel MPM, Handkiewicz Junak D, Lassmann M, Dietlein M, Luster M (2010). EANM procedure guidelines for therapy of benign thyroid disease. Eur J Nucl Med Mol Imaging.

[8] Nygaard B, Hegedüs L, Ulriksen P, Nielsen KG, Hansen JM (1999). Radioiodine therapy for multinodular toxic goiter. Arch Intern Med.

[9] Stokke C, Gabiña PM, Solný P, Cicone F, Sandström M, Gleisner KS (2017). Dosimetry-based treatment planning for molecular radiotherapy: a summary of the 2017 report from the Internal Dosimetry Task Force. EJNMMI Phys.

[10] Jönsson H, Mattsson S (2003). Single uptake measurement for absorbed dose planning for radioiodine treatment of hyperthyroidism. Cancer Biother Radiopharm.

[11] Perry WF, Hughes JFS (1952). The urinary excretion and thyroid uptake of iodine in renal disease. J Clin Invest.

[12] Hänscheid H, Lassmann M, Reiners C (2011). Dosimetry prior to I-131-therapy of benign thyroid disease. Z Med Phys.

[13] Reiners C, Schneider P (2002). Radioiodine therapy of thyroid autonomy. Eur J Nucl Med Mol Imaging.

[14] Health risks from exposure to low levels of ionizing radiation (2006). BEIR VII Phase 2.

[15] Mariani G, Tonacchera M, Grosso M, Orsolini F, Vitti P, Strauss HW (2021). The Role of Nuclear Medicine in the Clinical Management of Benign Thyroid Disorders, Part 1: Hyperthyroidism. J Nucl Med.

[16] Marinelli LD (1949). Dosage determination in the use of radioactive isotopes. J Clin Invest.

[17] Dirikoc A, Polat SB, Kandemir Z, Aydin C, Ozdemir D, Dellal FD (2015). Comparison of ultrasonography features and malignancy rate of toxic and nontoxic autonomous nodules: a preliminary study. Ann Nucl Med.

[18] Franklyn JA, Daykin J, Holder R, Sheppard MC (1995). Radioiodine therapy compared in patients with toxic nodular or Graves’ hyperthyroidism. QJM.

[19] Allahabadia A, Daykin J, Sheppard MC, Gough SC, Franklyn JA (2001). Radioiodine treatment of hyperthyroidism-prognostic factors for outcome. J Clin Endocrinol Metab.

[20] Tarantini B, Ciuoli C, Di Cairano G, Guarino E, Mazzucato P, Montanaro A (2006). Effectiveness of radioiodine (131-I) as definitive therapy in patients with autoimmune and non-autoimmune hyperthyroidism. J Endocrinol Invest.

[21] Lewis A, Atkinson B, Bell P, Courtney H, McCance D, Mullan K (2013). Outcome of 131I therapy in hyperthyroidism using a 550MBq fixed dose regimen. Ulster Med J.

[22] Gupta SK, McGrath S, Rogers K, Attia J, Lewis G, Viswanathan S (2010). Fixed dose (555 MBq; 15 mCi) radioiodine for the treatment of hyperthyroidism: outcome and its predictors. Intern Med J.

[23] Bonnema SJ, Hegedüs L (2012). Radioiodine therapy in benign thyroid diseases: effects, side effects, and factors affecting therapeutic outcome. Endocr Rev.

[24] van Isselt JW, de Klerk JMH, van Rijk PP, van Gils APG, Polman LJ, Kamphuis C (2003). Comparison of methods for thyroid volume estimation in patients with Graves’ disease. Eur J Nucl Med Mol Imaging.

[25] Stan MN, Durski JM, Brito JP, Bhagra S, Thapa P, Bahn RS (2013). Cohort study on radioactive iodine-induced hypothyroidism: implications for Graves’ ophthalmopathy and optimal timing for thyroid hormone assessment. Thyroid.

[26] Peters H, Fischer C, Bogner U, Reiners C, Schleusener H (1995). Radioiodine therapy of Graves’ hyperthyroidism: standard vs. calculated 131iodine activity. Results from a prospective, randomized, multicentre study. Eur J Clin Invest..

[27] Taprogge J, Gape PM, Carnegie-Peake L, Murray I, Gear JI, Leek F (2021). A systematic review and meta-analysis of the relationship between the radiation absorbed dose to the thyroid and response in patients treated with radioiodine for Graves’ disease. Thyroid..

