# Supplementary material for: Radioiodine treatment outcome by dosimetric parameters and renal function in hyperthyroidism
Source: Thyroid Res. 2022 Apr 25;15:8. doi: 10.1186/s13044-022-00126-4 (PMC9036725; doi:10.1186/s13044-022-00126-4)
Supplement: Supplementary file 1 — Additional file 1: Table S1. Comparison of outcome in the present study to articles in the literature that specified frequencies of eu-, hypo- and hyperthyroidism for both Nodular and Graves (nonsystematic review). The table is sorted by amount of activity. Figure S2. Graph of the data from Table 1 for the Graves cases. [file 13044_2022_126_MOESM1_ESM.pdf]

Table S1: Comparison of outcome in the present study to articles in the literature that specified frequencies of eu-, hypo- and hyperthyroidism for both Nodular and Graves (nonsystematic review). The table is sorted by amount of activity.

Study	Year	Type	N	Follow-up	Activity [MBq]	Outcome [%]				Comment
						Eu+Hypo	Eu	Hypo	Hyper	
1	2022	Nodular	569	3-12m	756	96	73	23	4	*MBq & outcome: weighted mean of multi- and uninodular; follow-up: range*
2	2011	Nodular	328	36m	647	79	67	12	21	*MBq: mean; outcome: article's mean for "TN/MNQ"; follow-up: mean*
3	2010	Nodular	300	4m	555	87	56	31	13	*MBq: fixed; outcome: weighted mean of article data for "TMNG" and "STA"*
4	2013	Nodular	75	12m	550	92	55	37	8	*MBq: fixed; outcome: for "multinodular"*
5	2006	Nodular	69	6m	465	75	58	17	25	*MBq & outcome: weighted mean of article data for "TA" and "TMG"*
5	2006	Nodular	69	36m	465	87	68	19	13	*MBq & outcome: weighted mean of article data for "TA" and "TMG"*
6	2004	Nodular	102	6m	370	72	63	9	28	*MBq: fixed; outcome: weighted mean of article data for "TMNG" and "TA"*
7	2001	Nodular	126	12m	269	71	40	32	29	*MBq: 185 or 370, weighted with a n=443/370-ratio as best guess (insufficient information; no response fr. auth.); outcome: for "toxic nodular"*
8	1995	Nodular	44	6m	185	66	55	11	34	*MBq: fixed; outcome: for "toxic nodular"*
3	2010	Graves	117	4m	555	62	15	46	39	*MBq: fixed*
4	2013	Graves	101	12m	550	89	11	78	11	*MBq: fixed*
5	2006	Graves	31	6m	533	84	10	74	16	*MBq: mean*
5	2006	Graves	31	36m	533	87	13	74	13	*MBq: mean*
1	2022	Graves	335	3-12m	468	79			22	*MBq: mean; follow-up: range*
2	2011	Graves	424	38m	462	74	28	46	26	*MBq & follow-up: mean*
6	2004	Graves	13	6m		69	23	46	31	*MBq: not specified*
7	2001	Graves	321	12m	269	70	15	55	31	*MBq: 185 or 370, weighted with a n=443/370-ratio as best guess (insufficient information; no response fr. auth.)*
8	1995	Graves	59	6m	185	44	17	27	56	*MBq: fixed*

1: Present study
2: Serdengecti and Sari, The outcome of I-131 therapy with fixed doses for Graves disease and toxic nodular/multinodular goiter in endemic region
3: Gupta et al, Fixed dose (555 MBq; 15 mCi) radioiodine for the treatment of hyperthyroidism: outcome and its predictors
4: Lewis et al, Outcome of 131I therapy in hyperthyroidism using a 550MBq fixed dose regimen
5: Tarantini et al, Effectiveness of radioiodine (131-I) as definitive therapy in patients with autoimmune and non-autoimmune hyperthyroidism
6: Erem et al, Radioiodine Treatment of Hyperthyroidism
7: Allahabadia et al, Radioiodine Treatment of Hyperthyroidism—Prognostic Factors for Outcome
8: Franklyn et al, Radioiodine therapy compared in patients with toxic nodular or Graves' hyperthyroidism

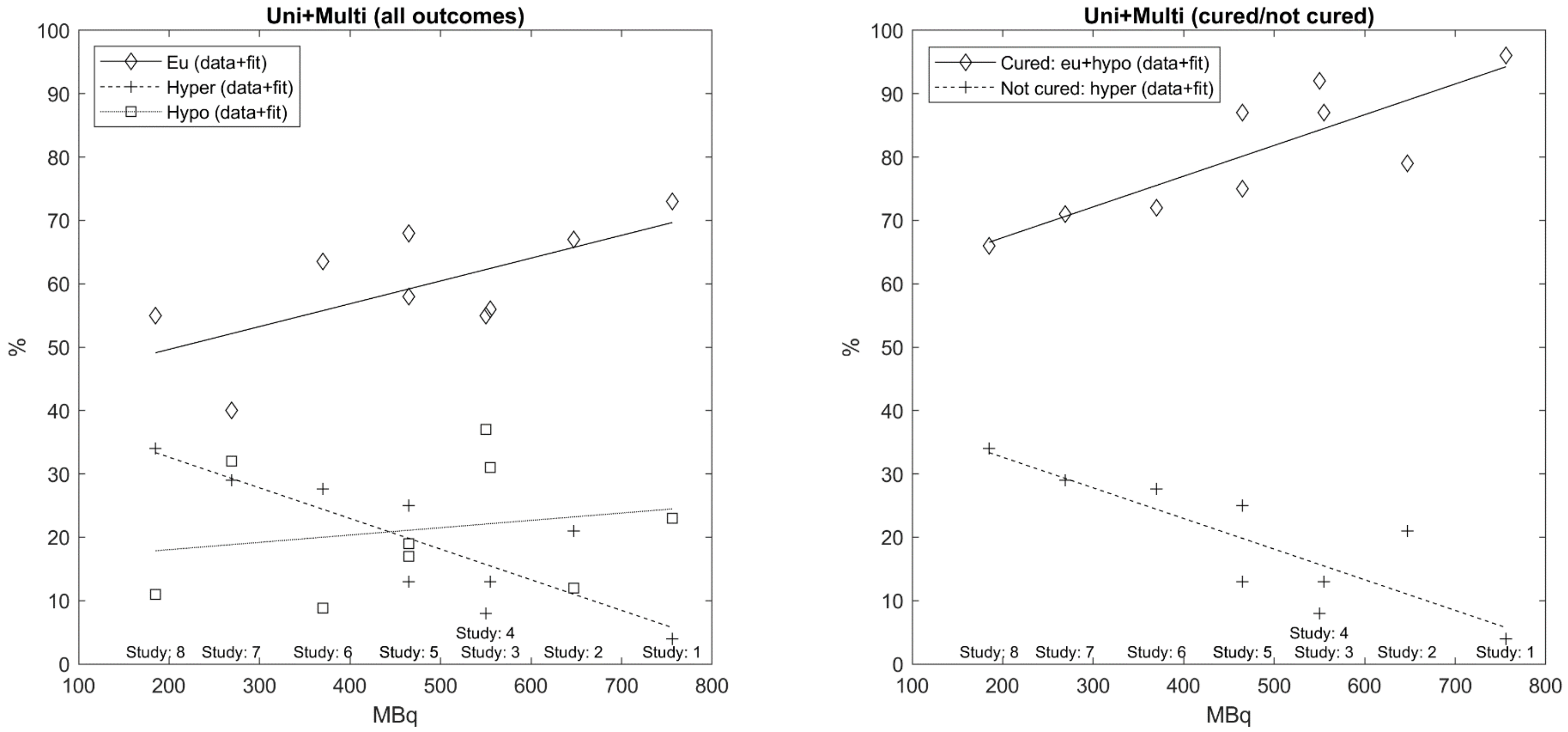

Figure S1: Graphs of the data from Table 1 for the nodular cases (uni+multi). Left graphs shows all three outcomes (eu, hyper and hypo) while the right graph shows cured (eu+hypo) and not cured (hyper).

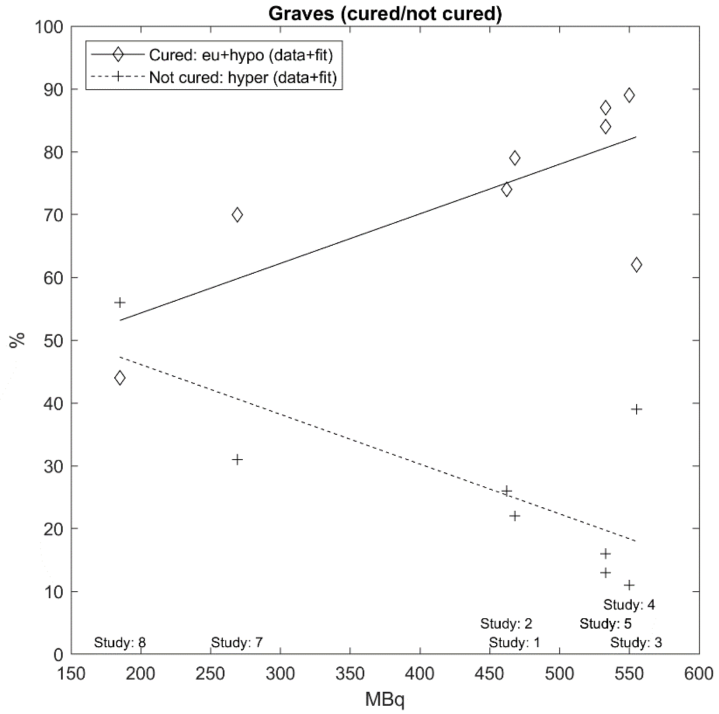

Figure S2: Graph of the data from Table 1 for the Graves cases.
